# DOES PHYSICAL FUNCTION RESPONSE TO INTENTIONAL WEIGHT LOSS IN OLDER ADULTS VARY BY RACE-ETHNICITY?

**DOI:** 10.1093/geroni/igz038.2512

**Published:** 2019-11-08

**Authors:** Daniel P Beavers, Rebecca Neiberg, Kristen Beavers, Dalane Kitzman, Barbara M Nicklas, Stephen Messier, Jack Rejeski, Stephen Kritchevsky

**Affiliations:** 1 Wake Forest School of Medicine, Winston-Salem, North Carolina, United States; 2 Wake Forest University, Winston-Salem, North Carolina, United States

## Abstract

The purpose of this study is to explore whether the effect of weight loss on physical function in older adults varies by race/ethnicity. Individual level data from 1369 older, (67.7±5.4 years), obese (BMI: 33.9±4.4 kg/m2), adults (30% male, 21% African American) who participated in eight randomized controlled trials of weight loss were pooled. Studies were 5-6 months in duration and collected baseline demographic and pre/post gait speed (n=1296), short physical performance battery (SPPB; n=866), and grip strength (n=401) data. Treatment effects were generated by weight loss assignment [weight loss (WL; n=764) versus non-weight loss (NWL; n=605)], as well as categorical amount of weight change (high loss: >-7%, moderate loss: -7 to -3%, and weight gain/stability: <-3%). Analyses were adjusted for age, sex/gender, study, education, baseline BMI, and baseline value of the outcome measure of interest. Race/ethnicity stratified results were presented if the interaction term was p≤0.10. A race/ethnicity*weight loss assignment interaction was observed for gait speed (p=0.07), with African Americans experiencing greater weight loss-associated improvement (WL: 0.07±0.01 m/s versus NWL: 0.02±0.01 m/s; p=0.03) compared to Whites (WL: 0.08±0.01 m/s versus NWL: 0.07±0.01 m/s). A race/ethnicity*weight loss amount interaction was also observed for gait speed (p<0.01), with greater weight loss associated with greater improvement in both African Americans and Whites; although, gains were most apparent in African Americans experiencing high loss (0.12±0.02 m/s) compared to gain/stability (0.01±0.01 m/s). The beneficial effects of weight loss on gait speed appear greater in African Americans and are augmented with greater weight loss.

